# Decline of medical student idealism in the first and second year of medical school: a survey of pre-clinical medical students at one institution

**DOI:** 10.3402/meo.v18i0.21194

**Published:** 2013-08-21

**Authors:** Christopher P. Morley, Carrie Roseamelia, Jordan A. Smith, Ana L. Villarreal

**Affiliations:** 1 Department of Family Medicine, SUNY Upstate Medical University, Syracuse, NY, USA; 2 Department of Public Health and Preventive Medicine, SUNY Upstate Medical University, Syracuse, NY, USA; 3 Department of Psychiatry and Behavioral Sciences, SUNY Upstate Medical University, Syracuse, NY, USA; 4 GTx, Inc., Memphis, TN, USA

**Keywords:** medical students, idealism, surveys, career choice

## Abstract

**Background:**

Idealism declines in medical students over the course of training, with some studies identifying the beginning of the decline in year 3 of US curricula.

**Purposes:**

This study tested the hypothesis that a decline in medical student idealism is detectable in the first two years of medical school.

**Methods:**

We sought to identify differences in survey responses between first-year (MS1) and second-year (MS2) medical students at the beginning (T1) and end (T2) of academic year 2010 on three proxies for idealism, including items asking about: (a) motivations for pursuing a medical career; (b) specialty choice; and (c) attitudes toward primary care. Principle component analysis was used to extract linear composite variables (LCVs) from responses to each group of questions; linear regression was then used to test the effect of on each LCV, controlling for race, ethnicity, rural or urban origins, gender, and marital status.

**Results:**

MS2s placed more emphasis on status/income concerns (*β*=0.153, *p*<0.001), and much less emphasis on idealism as a motivator (*β*=−0.081, *p*=0.054), in pursuing a medical career; more likely to consider lifestyle and family considerations (*β*=0.098, *p*=0.023), and less likely to consider idealistic motivations (*β*=−0.066, *p*=NS); and were more likely to endorse both negative/antagonistic (*β*=0.122, *p*=0.004) and negative/sympathetic (*β*=0.126, *p*=0.004) attitudes toward primary care.

**Conclusions:**

The results are suggestive that idealism decline begins earlier than noted in other studies, implying a need for curricular interventions in the first two years of medical school.

## Background

Acceptance into medical school often hinges on students having ‘the right stuff’, which includes an attitude deemed necessary for physicians to run an actual practice (1, 2). Part of this ‘right’ attitude or ideology includes a sense of idealism toward medical practice and patient care. However, a number of studies have found that students tend to lose empathic and idealistic motivations over the course of medical education (1, 3–15). This loss of idealism includes a decreased interest in working in underserved communities, feeling of less responsibility for the health of society as a whole, and increased jadedness toward the medical profession overall (4, 5).

While it is difficult to pinpoint the shift in student's thought throughout their medical education, there are a number of factors that have been recognized to correlate with the current movement. Students are more inclined to prioritize lifestyle choices when deciding on a medical career tract and tend to be dissuaded from primary care due to perceived long hours and lower income capabilities as compared to other specialties (16, 17). Burn-out among medical students may play a substantial role (18–21). For example, Enoch and colleagues have reported that higher levels of burn-out among students were associated with an increase in gravitation toward specialties with greater lifestyle control and higher income.

Role modeling and faculty teaching are also likely to have an effect on student's idealism and perceptions of various disciplines (22). For instance, a decline in idealism may be a contributory factor in the shift away from the selection of primary care specialties and careers by graduating medical students. As Newton has noted, the downward shift in idealism may have the most impact upon male students who choose non-core medical specialties (11). A study conducted by Holmes in 2008 revealed that students reported faculty making devaluing or derogatory comments about family medicine in their lectures and interactions, a practice commonly referred to as ‘bashing’. It was also noted that residents as well as other students bashed the practice of family medicine as well (23). In this sense, a ‘hidden curriculum’ (24) may be behind the idealistic shift. Hafferty defines hidden curriculum as cultural influences that are transmitted at the organizational or structural levels (25). These influences are often not outwardly acknowledged by the institution or the faculty, but can have a profound effect on medical student attitude and mentality toward practice (22). Finally, empathy in students has also been noted to drop over the course of training (1, 11, 26), although at least one study has indicated that empathy might spike in year two, before a precipitous drop during clinical years (27). While empathy is relevant to a physician's relationships with individual patients, and idealism typically refers to the physician's overall goal for medical practice, it is possible that the observable declines in each domain over the course of medical training are linked to common factors, or in fact, influence each other.

## Purposes

There is some suggestion that the fall-off in idealism occurs after the second year (12) of medical school, beginning during the third year of medical school (6, 8) or later. For this study, we compare first-year (MS1) and second-year (MS2) medical students in three dimensions of idealism in a medical context, using data from a survey conducted at the beginning and end of academic year 2010–2011 (AY2010). We asked about the reasons students decided to pursue a career in medicine, about what they were thinking about in terms of a future specialty, and about student attitudes toward primary care. Although these particular items have not been used to directly measure idealism in the past, the current instrument asked about motivating factors behind these choices and attitudes, discerning between apparent self-interested considerations such as financial reward, prestige, and lifestyle, and apparent external considerations, such as a desire to serve one's community. As no single standard for the measurement of medical student idealism exists, the ability to extract factors that contrast financial motivation from community service orientation, for example, provide what we believe to be a meaningful perspective on the construct.

## Methods

This study utilized data collected via a student survey, distributed at two time points in AY2010 – in the first month of the year and again at the end of AY2010. The survey was administered to both first-year (MS1) and second-year (MS2) medical students, creating four cohort/time points: MS1 at T1, MS1 at T2, MS2 at T1, and MS2 at T2. For these analyses, we sought to identify whether there were detectable differences in survey responses between the two groups at the two time points on three matrix questions, selected to represent three dimensions of idealism:

- How important are the following factors in considering your career in medicine?
- How important are the following factors in considering your choice for a specialty?
- Please indicate how much you agree or disagree with the following statements (indicative of attitudes about primary care).

### Context

The survey was conducted at an allopathic medical school in the northeastern region of the United States that admits approximately 160 students per year into its medical doctor (MD) program. Students follow a traditional curriculum, with the first two years of the four-year program devoted to basic science coursework and a two-year long clinical skills course, with little-to-no patient contact. The second two years are devoted to clinical training through required clerkships and electives. The survey was administered on paper during meetings of the clinical skills course.

### Survey development

The survey instrument was purposively constructed to gauge pre-clinical student interests and attitudes toward specific specialties, career paths, and types and contexts of service. The instrument relied principally upon matrix questions, with individual items rated on Likert scales. A beta test period and group was not available, so the survey was designed with questions on similar concepts interspersed throughout the instrument. This allowed for *post-hoc* calculation of Cronbach's α following the first administration of the instrument. The instrument demonstrated very good reliability between related items, such as between questions related to primary care interest (Cronbach's α=0.838) and to income expectations (Cronbach's α=0.797). Due to privacy concerns, data points about each student were limited to gender, marital status, number of children, race and ethnicity, and zip code of high school from which they graduated (to allow for approximation of rural/urban upbringing). Age was not included, because the student population receiving the survey is fairly homogenous in terms of age, and age outliers would have been identifiable.

### Survey implementation

The paper survey was distributed during a mandatory clinical skills course for MS1s and MS2s. The distribution occurred for all students once in August of 2010, at the beginning of the AY2010, and a second time, in May 2011, at the end of the AY2010. The cognizant institutional review board (US IRB Registration #00000391, Federal-Wide Assurance #00005967) recognized this study as exempt from review, because of the delinking of responses from identities, and because of the minimal risk associated with participation. Students were verbally informed about the purpose of the survey, that their participation was voluntary, and that their identities would not be linked to their responses.

### Analysis

**Fig. 1:**
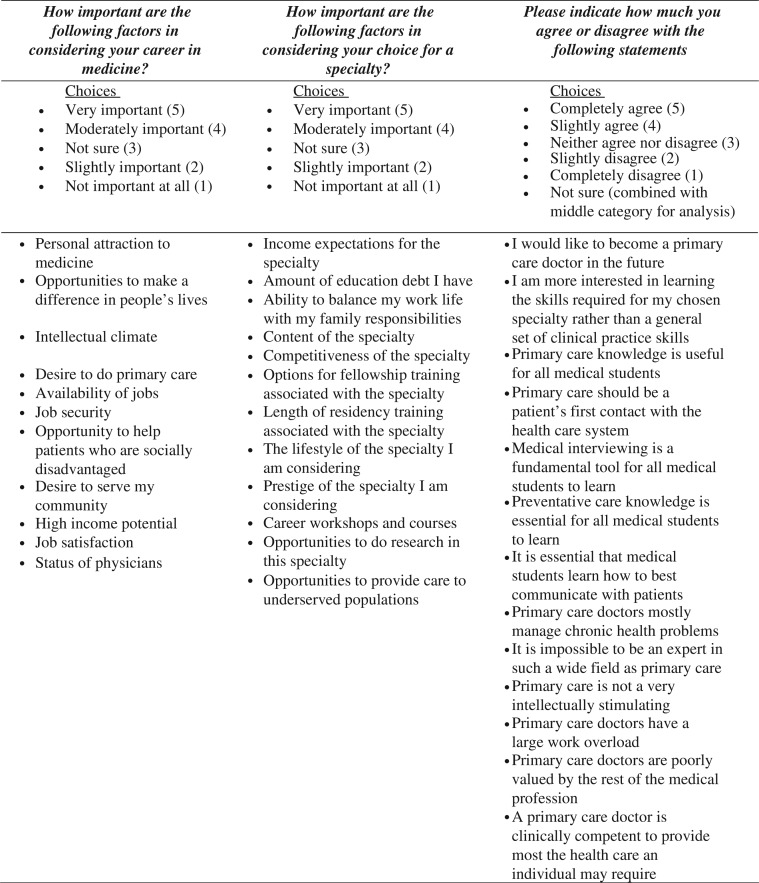
Matrix questions and items used.

The specific items were ranked on 5-item Likert scales, ranging from ‘Not important at all’ to ‘Very important’ for the medicine and specialty questions, and a 6-point Likert scale ranging from ‘Completely disagree’ to ‘Completely agree’, with ‘Neither agree nor disagree’ as a central anchor, and an additional ‘Not sure’ option, for the matrix of primary care attitudinal statements. Responses were scaled from 1 (Not important at all/completely disagree) to 5 (Very important/completely agree). Responses on the primary care statements marked ‘Not sure’ were incorporated into the neutral anchor category (coded as 3), so that all items were analyzed on a five-point scale. The three matrix questions and items are further described in Fig. 1.

To answer the central questions, representing alternative constructs of idealism, the analysis was conducted in four steps, with all analyses conducted in SPSS v21.

The individual items under each of the three questions were compared across the four response points directly. Given the ordinal nature of the data, we utilized the Kruskal–Wallis test to assess significance of any differences.

We then identified latent factors and created linear composite variables (LCVs) for the items under each question via principal component analysis (PCA), extracting factors that exceeded an eigenvalue of 1, and assessing solutions after varimax rotation. Each factor was named based upon the top factor loadings, using a threshold of 0.700 as indicative of a major component and 0.400 as a minor component. The factors were saved as LCVs in the data set, each with a mean of 0 and a standard deviation of 1.

The groups were then compared using analysis of variance (ANOVA) across the LCVs extracted through PCA to assess significance of any observed differences between mean factor scores for MS1s and MS2s and across the time points.

Each factor was then modeled as a product of year and time point controlling for potential confounders. A variable representing MS year and time point was entered, with covariates removed via backward stepwise ordinary least squares (OLS) linear regression procedure. Each predictor was entered as a variable in models following the form:

$$\begin{array}{l} {\text{Factor}=\text{Constant}+\beta }_{1}{\text{MS}2/\text{T}2+\beta }_{\text{k}\ldots I}\text{Covariates}\\ \text{Year}/\text{Time}:\text{MS}1\text{at T}1=1(\text{reference});\text{MS}1\text{at}\\ \text{T}2=\text{MS}2\text{at T}1=3,\text{MS}2\text{at T}2=4\\ \text{Race}:\text{White}/\text{Caucasian}=1/\text{Non}-\text{White}=0\\ \text{Ethnicity}:\text{Hispanic}=1/\text{Not Hispanic}=0 \end{array}$$

Rural/urban: students originally from rural areas were determined by using the Rural–Urban Commuting Area (RUCA) approximations based upon the zip code where the student attended secondary school (28). Two dummy variables were created

$$\begin{array}{c} \text{Rural}=1,\text{Non}-\text{Rural}=0\\ \text{Urban}=1,\text{Non}-\text{Urban}=0\\ \text{Marital Status}:\text{Married}=1/\text{Not Married}=0 \end{array}$$

## Results

A total of 596 responses were included in the analysis, with response rates at each time point ranging from 87.5 to 99.4% (out of a class size of 160).The two cohorts were very similar, with significant differences only between the number of self-identified Hispanic students in MS1 (MS1=7, MS2=1; χ^2^=8.110, *p*=0.017), and more married students in MS2 (MS1=6, MS2=23; χ^2^=11.724, *p*=0.001). Additionally, the student body is more white (63.1% vs. 54.6% nationally in 2012; *p*=0.001) and male (58.0% vs. 51.6% nationally in 2012; *p*<0.001) than the nation as a whole (29). Further detail regarding the sample is available in Table 1.

**Table 1:** Demographics of the sample, by group

	MS1 T1 (n=159)	MS1 T2 (n=150)	MS2 T1 (n=140)	MS2 T2 (n=147)
Gender				
Male	79	79	70	74
Female	63	63	65	66
Race				
White	103	98	87	94
Black/African American	24	22	22	25
Asian	29	26	23	26
Native American	0	1	0	2
Others	2	0	0	0
Unknown	1	0	0	6
Hispanic				
Yes	7	9	1	1
No	150	138	133	144
Attended high school in the United States				
Yes	146	139	119	132
No	12	11	15	15
Marital status				
Single	151	141	107	119
Married	6	6	23	23
Divorce	1	2	3	3
Unknown	0	0	1	1
Rural origins				
Non-rural	132	124	102	115
Rural	14	15	16	16

MS1=First-Year Medical Students; T1=beginning of the academic year; MS2=Second-Year Medical Students; T2=end of the academic year.

In response to the question, ‘How important are the following factors in considering your career in medicine?’, respondents from the MS1 cohort placed significantly higher importance on ‘(d)esire to serve my community’ (*p*=0.023). Conversely, respondents from the MS2 cohort placed significantly more importance on ‘(h)igh income potential’ (*p*=0.006). Similarly, MS1s were slightly more likely to place importance on ‘(a)vailability of jobs’ (*p*=0.034). The results are displayed in Table 2.

**Table 2:** Comparison of MS1 and MS2 on importance of factors in considering medicine

Items	MS1 T1	MS1 T2	MS2 T2	MS2 T2	*p*
**How important are the following factors in considering your career in medicine?**					
Personal attraction to medicine	4.83	4.81	4.82	4.81	NS
Opportunities to make a difference in peoples’ lives	4.88	4.81	4.84	4.83	NS
Intellectual climate	4.45	4.50	4.52	4.46	NS
Desire to do primary care	3.03	2.87	2.72	2.73	NS
Availability of jobs	3.58	3.68	3.88	3.87	0.034*
Job security	3.81	3.99	4.04	4.03	NS
Opportunity to help patients who are socially disadvantaged	4.13	3.99	3.90	3.90	0.074
Desire to serve my community	4.42	4.24	4.35	4.36	0.023*
High income potential	2.80	3.09	3.33	3.30	0
Job satisfaction	4.76	4.70	4.79	4.80	NS
Status of physicians	2.69	2.81	2.93	2.83	NS
					
**How important are the following factors in considering your choice for a specialty?**					
Income expectations for the specialty	2.80	3.09	3.21	3.20	0.004**
Amount of education debt I have	2.79	3.16	2.98	2.95	NS
Ability to balance my work life with my family responsibilities	4.41	4.33	4.63	4.61	0.001**
Content of the specialty	4.73	4.65	4.7	4.68	NS
Competitiveness of the specialty	3.16	3.17	3.05	3.05	NS
Options for fellowship training associated with the specialty	3.29	3.2	3.45	3.38	NS
Length of residency training associated with the specialty	3.17	3.24	3.42	3.45	0.05*
The lifestyle of the specialty I am considering	4.22	4.19	4.41	4.37	0.015*
Prestige of the specialty I am considering	2.18	2.52	2.46	2.39	NS
Career workshops and courses	2.73	2.51	2.53	2.47	NS
Opportunities to do research in this specialty	2.60	2.61	2.44	2.39	NS
Opportunities to provide care to underserved populations	3.42	3.3	3.3	3.28	NS
					
**Attitudes toward primary care**					
I would like to become a primary care doctor in the future	3.14	3.04	2.93	2.96	NS
I am more interested in learning the skills required for my chosen specialty rather than a general set of clinical practice skills	2.78	2.97	2.85	2.81	NS
Primary care knowledge is useful for all medical students	4.76	4.59	4.67	4.67	NS
Primary care should be a patient's first contact with the health care system	4.40	4.37	4.36	4.37	NS
Medical interviewing is a fundamental tool for all medical students to learn	4.88	4.81	4.83	4.84	NS
Preventative care knowledge is essential for all medical students to learn	4.81	4.73	4.50	4.53	<0.001**
It is essential that medical students learn how to best communicate with patients	4.88	4.84	4.87	4.86	NS
Primary care doctors mostly manage chronic health problems	3.22	3.32	3.75	3.78	<0.001**
It is impossible to be an expert in such a wide field as primary care	2.73	2.81	2.98	3.01	0.085
Primary care is not very intellectually stimulating	1.89	2.04	2.2	2.21	0.009**
Primary care doctors have a large work overload	3.60	3.60	3.95	3.95	0.001**
Primary care doctors are poorly valued by the rest of the medical profession	3.47	3.43	3.81	3.78	0.015*
A primary care doctor is clinically competent to provide most the health care an individual may require	3.99	3.90	4.02	4.01	NS

* Significant at the 0.05 level

** Significant at the 0.01 level

Likert Scale (1=‘Not important at all’; 5=‘Very Important’). Differences tested via Kruskall–Wallis test; *p* below 0.10 displayed; NS=Not Significant.

In response to the question, ‘How important are the following factors in considering your choice for a specialty?’, respondents from the MS2 cohort placed significantly higher importance on ‘(i)ncome expectations for the specialty’ (*p*=0.004) and on the ‘(a)bility to balance my work life with my family responsibilities’ (*p*=0.001), as well as on the ‘(l)ength of residency training associated with the specialty’ (*p*=0.05). The ‘lifestyle of the specialty I am considering’ was also rated more highly by MS2s(*p*=0.015). The results are displayed in Table 2.

Attitudes toward primary care topics and issues were also split between the two cohorts. MS1s were in greater agreement with the statement that ‘(p)reventive care knowledge is essential for all medical students to learn’ (*p*<0.001). However, MS2s were in greater agreement with statements that were more negative toward primary care, including ‘(p)rimary care doctors mostly manage chronic health problems’ (*p*<0.001), ‘(i)t is impossible to be an expert in such a wide field as primary care’ (*p*=0.022), ‘(p)rimary care is not very intellectually stimulating’ (*p*=0.009), ‘(p)rimary care doctors have a large workload’ (*p*<0.001), and ‘primary care doctors are poorly valued by the rest of the medical profession’ (marginally significant at *p*=0.015). The results are further displayed in Table 2.

Table 2 additionally illustrates that similar trends measured across the four measurement points were observed across each response, with gradually increasing emphasis on economic, lifestyle, and career considerations, and decreasing emphasis on service to underserved populations, the community, and primary care.

PCAs for each of the three item matrices extracted four linear, composite factors in each matrix, as shown in Table 3. Comparisons across MS1s and MS2s at the two time points on each LCV are displayed in Table 4. MS1s were more likely to consider idealism as a motivator to pursue medicine (approaching significance at *p*=0.064). Conversely, MS2s were more concerned with employment and job security (approaching significance at *p*=0.070) as well as status and income (approaching significance at *p*=0.061) in choosing to pursue a medical career. MS2s were also significantly more likely to consider lifestyle and family when thinking about specialty choices (*p*=0.002), but were less likely to display idealism in specialty considerations (not significant). MS2s also had substantially more negative attitudes toward primary care. These included what we interpreted to be negative and antagonistic views (*p*<0.001), as well as negative but sympathetic views of primary care (*p*<0.001).

**Table 3:** Linear composite variables (LCVs) derived via principal component analysis, with varimax rotation

Factor (% of variance)	Items (component score)
**How important are the following factors in considering your career in medicine?**	
Employment and job security (18.300)	Job security (**0.891**)
	Availability of jobs (**0.886**)
	High income potential (0.419)
	Intellectual Climate (0.402)
Idealism in medicine (17.713)	Desire to serve my community (**0.753**)
	Opportunity to help patients who are socially disadvantaged (**0.722**)
	Desire to do primary care (0.673)
	Opportunities to make a difference in peoples’ lives (0.586)
Attraction to medicine (14.013)	Personal attraction to medicine (**0.788**)
	Job satisfaction (**0.726**)
	Intellectual climate (0.403)
Status and income (13.384)	Status of physicians (**0.907**)
	High income potential (**0.712**)
	
**How important are the following factors in considering your choice for a specialty?**	
Prestige and income	Competitiveness of the specialty (**0.754**)
	Prestige of the specialty I am considering (0.699)
	Options for fellowship training associated with the specialty (0.668)
	Income expectations for the specialty (0.536)
	Career workshops and courses (0.502)
	Opportunities to do research in this specialty (0.470)
Lifestyle and family	The lifestyle of the specialty I am considering (**0.821**)
	Ability to balance my work life with my family responsibilities (**0.798**)
	Length of residency training associated with the specialty (0.539)
Idealism in specialty choice	Opportunities to provide care to underserved populations (**0.823**)
	Career workshops and courses (0.559)
	Opportunities to do research in this specialty (0.488)
Debt over content interest	Content of the specialty (**−0.0786**)
	Amount of education debt I have (0.578)
	Income expectations for the specialty (0.448)
	
**Attitudes toward primary care**	
Value of primary care skills	Medical interviewing is a fundamental tool for all medical students to learn (**0.873)**
	It is essential that medical students learn how to best communicate with patients (**0.837**)
	Primary care knowledge is useful for all medical students (**0.726**)
	Preventative care knowledge is essential for all medical students to learn (**0.707**)
Considering PC career	I would like to become a primary care doctor in the future (0.667)
	Primary care should be a patient's first contact with the health care system (0.631)
	A primary care doctor is clinically competent to provide most the health care an individual may require (0.568)
	Primary care is not a very intellectually stimulating (−0.565)
Negative/antagonistic view of PC	Primary care doctors mostly manage chronic health problems (**0.796**)
	It is impossible to be an expert in such a wide field as primary care (**0.753**)
	I am more interested in learning the skills required for my chosen specialty rather than a general set of clinical practice skills (0.494)
Negative/sympathetic view of PC	Primary care doctors are poorly valued by the rest of the medical profession (**0.804**)
	Primary care doctors have a large work overload (**0.788**)

EmpMajor components (≥0.700) are listed in bold; minor components are listed in roman (≥0.400).

**Table 4:** Distribution of factor scores across groups and time points

Factors	MS1 T1	MS1 T2	MS2 T1	MS2 T2	*p*
Employment and job security	−0.17	0.00	0.10	0.09	0.070
Idealism in medicine	0.18	−0.06	−0.07	−0.07	0.064
Attraction to medicine	0.02	−0.07	0.03	0.01	NS
Status and income	−0.15	−0.04	0.14	0.08	0.061
Prestige and income	−0.03	0.04	0.02	−0.02	NS
Lifestyle and family	−0.12	−0.18	0.18	0.16	0.002
Idealism in specialty choice	0.13	−0.03	−0.04	−0.07	NS
Debt over content interest	−0.14	0.13	0.01	0.01	NS
Value of PC skills	0.10	−0.07	−0.03	−0.01	NS
Considering PC career	0.12	0.03	−0.10	−0.08	NS
Negative/antagonistic view of PC	−0.24	−0.13	0.18	0.21	<0.001
Negative/sympathetic view of PC	−0.19	−0.20	0.22	0.20	<0.001

Differences across groups measured via ANOVA.

NS=Not Significant.

OLS regression analyses of the effect of each MS/T point on each LCV revealed similar patterns. For importance of factors in considering a medical career, a nearly-significant negative trend in ‘Idealism in medicine’ was observed across MS/T time points, indicating a reduction in this factor across temporally progressive measurement points (*β*=−0.081, *p*=0.054). Conversely, the factor representing ‘Status and income’ increased in importance across measurements (*β*=0.153, *p*<0.001).

Similarly, MS2s at T2 were more likely to consider lifestyle and family considerations (*β*=0.098, *p*=0.023), and less likely to consider idealistic motivations (*β*=−0.066, *p*=NS), when considering specialties to enter. MS2s were also more likely to endorse both negative/antagonistic (*β*=0.122, *p*=0.004) and negative/sympathetic (*β*=0.126, *p*=0.004) attitudes toward primary care. All analyses controlled for race, ethnicity, rural or urban origins, gender, and marital status, either through inclusion within the final model, or through backward elimination. The results are displayed with greater detail in Table 5.

**Table 5:** Results of backward stepwise linear regression analyses of each factor, modeled^ as an outcome of MS2/T2

Factors	Predictors	*β*(sig)	Model summary
**How important are the following factors in considering your career in medicine?**			
Employment and job security	Year and test	0.069 (0.111)	*R* ^2^=0.021
	Rural/urban (rural=1)	−0.381 (0.010)**	*F*=4.393 (0.013)
Idealism in medicine	Year and test	−0.081 (0.054)	*R* ^2^=0.075
	White/Caucasian	−0.587 (<0.001)**	*F*=16.970 (<0.001)
Attraction to medicine	Year and test	0.018 (0.659)	
	White/Caucasian	0.431 (<0.001)**	*R* ^2^=0.059
	Gender	0.278 (0.003)**	*F*=6.501 (<0.001)
	Married	−0.345 (0.029)*	
Status and income	Year and test	0.153 (<0.001)**	
	Gender	−0.383 (<0.001)**	*R* ^2^=0.082
	Rural/urban	0.355 (0.013)*	*F*=9.316 (<0.001)
	Married	−0.354 (0.027)*	
			
**How important are the following factors in considering your choice for a specialty?**			
Career and prestige	Year and test	0.067 (0.121)	*R* ^2^=0.033
	Married	−0.608 (<0.001)**	*F*=7.156 (0.001)
Lifestyle and family	Year and test	0.098 (0.023)*	*R* ^2^=0.035
	Gender	0.288 (0.003)**	*F*=7.456 (0.001)
Idealism in specialty choice	Year and test	−0.066 (0.126)	*R* ^2^=0.051
	White/Caucasian	−0.402 (<0.001)**	*F*=7.342 (<0.001)
	Gender	0.205 (0.040)*	
Debt over content interest	Year and test	0.009 (0.837)	*R* ^2^=0.053
	White/Caucasian	−0.422 (<0.001)**	*F*=7.644 (<0.001)
	Married	0.405 (0.012)*	
			
**Attitudes toward primary care**			
Value of PC skills	Year and test	−0.055 (0.214)	*R* ^2^=0.018
	Gender	0.253 (0.013)*	*F*=3.703 (0.025)
Considering PC career	Year and test	−0.063 (0.151)	*R* ^2^=0.033
	White/Caucasian	−0.363 (0.001)**	*F*=4.642 (0.003)
	Rural/Urban	0.317 (0.043)*	
Negative/antagonistic view of PC	Year and test	0.122 (0.004)**	*R* ^2^=0.079
	White/Caucasian	−0.422 (<0.001)**	*F*=11.765 (<0.001)
	Rural/Urban	−0.349 (0.019)*	
Negative/sympathetic view of PC	Year and test	0.126 (0.004)**	*R* ^2^=0.040
	Married	0.396 (0.017)*	*F*=8.672 (<0.001)

* Significant at the 0.05 level

** significant at the 0.01 level.

^ Controlling for race (White=1), ethnicity (Hispanic=1), rural or urban (1/0) origin, gender (female=1), marital status (married=1). Predictors displayed are those left in the final model produced by backward stepwise entry of covariates.

## Conclusions

This study attempted to measure differences between MS1s and MS2s at the beginning and end of the academic year on three dimensions of idealism, at one medical school. The results are highly suggestive that a decline in idealism may begin earlier than noted in some studies, and adds to the literature by: (a) providing additional and current evidence for declines in idealism; and (b) by adding evidence that such declines begin early in medical training. Individually significant measurements tell part of the story, but the trends across all of the variables included here tell a similar story, which is not simply that one variable representing a construct of ‘Idealism’ decreases over time; but rather, that several items that represent idealistic motivations for career direction decrease as medical careers move forward temporally, and concurrently measured items representing concerns over money, lifestyle, career, and prestige increase.

The observations described above were made at a medical school with what might be considered a ‘traditional’ US medical curriculum, with the first two years consisting of intensive basic science coursework, a basic ‘doctoring’ course (the one used to disseminate the survey described here), and little-to-no direct clinical exposure to patients. We therefore believe these results may generalize to other US medical schools.

### Weaknesses

This study has a number of limitations and weaknesses. The main issue is that the data we compared were from two different student cohorts. A better approach would be to track attitudinal changes in individual students over time, as done by previous researchers (6, 12). In this case, the cognizant institutional review board disallowed the use of individual identifiers, thereby eliminating the possibility of connecting responses from individuals over time. However, there were no substantial changes to the curriculum or to admission requirements between the two years, and the groups were demographically comparable. The need to maintain the anonymity of the data also precluded use of age as a variable, as outliers would have been easily identified. It is entirely possible that age may play a role in any reduction in idealism.

As noted previously, the sample used in this study was more white and male than some measures of the US medical student population nationally. This may be an important issue, as ‘white’ and ‘female’ covariates were significant in some of the multivariate models presented here. However, at no point in our analyses did either variable erase an otherwise-significant finding for the MS2 variable, and in fact, the MS2-at-T2 variable was robust from individual item comparisons, through bivariate comparisons of the LCVs derived from PCA procedures, to the controlled regression models employed in the final analytic step. A related issue is that this was a single-institution study.

An additional weakness may lie in the instrument used to collect data. The instrument was developed quickly for deployment in time for the beginning of a school year, and it was designed with general tracking of student interests in mind. We did not use a standardized instrument to test idealism, or related constructs like empathy. The analyses presented here should therefore be considered secondary analyses of an existing data set. However, the instrument appears to be internally consistent and reliable. Additionally, the study design utilizes interest in and attitudes toward primary care as one proxy for idealism, based upon the assumption that medical students interested in primary care are cognizant of the generally lower compensation within Family Medicine, Pediatrics, and General Internal Medicine, relative to some specialties, and are hence motivated less by compensation. It is not only possible, but likely, that many students with interests in other core specialties (e.g., Psychiatry, General Surgery) are equally as idealistic as those inclined to pursue primary care specialties. Using primary care attitudes as one dimension of or proxy for idealism is therefore imperfect; however, we do view it as valid, especially when used in conjunction with other dimensions, as we have presented here.

### Summary

This study suggests that idealism in medical students begins to decline in the first two years of medical school. The decline may be partially due to a hidden curriculum that shifts students away from relatively less lucrative and more service-oriented careers. It is also possible that students become cognizant of their level of rising debt during this time period (30). Additionally, selection of high-achieving applicants based primarily on Medical College Aptitude Test (MCAT) scores and undergraduate grade point average may overlook other dimensions of medical aptitude during the admissions process. However, this study did not explore the reasons for the apparent decline in idealism, as measured here. Additionally, a number of targets for intervention exist. For example, dedicated curricula, such as the online curriculum described by Wiecha and Markuns (31), may be effective. Concerted efforts to develop interventions that increase or maintain a constellation of related desirable characteristics represented by idealism, empathy, professionalism (32–34), and humanism (31) while decreasing burnout, along with rigorous evaluation and continual improvement of such interventions, may be warranted. Service learning (35) in community (36, 37) or global health contexts (38) may represent additional or alternative interventions. In any case, the present study does suggest the need for earlier intervention (31) for the preservation of idealism in medical students, if we are to train an adequate number of primary care and underserved-focused physicians going forward.
